# Rules for Good Health

**Published:** 1889-11

**Authors:** 


### Rules for Good Health.

Be regular in your habits.

If possible go to bed at the same hour every night.

Rise in the morning soon after you are awake.

A sponge bath of cold or tepid water should be followed by friction with towel or hand.

Eat plain food.

Begin your morning meal with fruit.

Don't go to work immediately after eating.

Be moderate in the use of liquids at all seasons.

It is safer to filter and boil drinking water.

Exercise in the open air whenever the weather permits.

In malarious districts do your walking in the middle of the day.

Keep the feet comfortable and well protected.

Wear woollen clothing the year round.

See that your sleeping rooms and living rooms are well ventilated, and that sewer gas does not enter them.

Brush your teeth at least twice a day, night and morning.

Don't worry, it interferes with the healthful action of the stomach.

You must have interesting occupation in vigorous old age. Continue to keep the brain active. Rest means rust.—*Herald of Health.*

---

THE PHYSICIAN'S FEE.—A decision was recently given in a suit for payment for services brought by Dr. Langeley of New York, so says the New York *Medical Record.* Judge Brady, of the New York Supreme Court, decided that in an action by a surgeon for professional services, the plaintiff has a right to show that his standing in the profession is high as bearing upon the question of the measure of his compensation. The judge further said: "There is also evidence tending to establish a custom or rule of guidance as to charges of physicians for services rendered, and which makes the amount dependent upon the means of the patient—his financial ability or condition. This is a benevolent practice which does not affect the abstract question of value nor impose any legal obligations to adopt it, and can not be said to be universal. Indeed, there does not seem to be any standard by which, in the application of the rule, the amount to be paid can be ascertained. Each case is under the special disposition of the surgeon or physician attending, and he is to decide as to the reduction to be made on account of the circumstances of his patient; and therefore, when the amount is in dispute, it follows that it is to be determined by proofs to be given on either side. The measure of compensation must be controlled more or less by ability in all the professions, and the service rendered by its responsibilities and success.—*Cincinnati Med. News.*

